# Association between cervical length and massive intraoperative bleeding in patients with suspected placenta accreta spectrum combined with placenta previa: A retrospective cohort study

**DOI:** 10.3389/fsurg.2022.1028494

**Published:** 2022-10-21

**Authors:** Fusen Huang, Jingjie Wang, Yi Xu, Qiuju Xiong, Wenjian Wang, Jia Zhuo, Qiuling Xia, Xiaojuan Yang

**Affiliations:** ^1^ Department of Anesthesiology, First Affiliated Hospital of Chongqing Medical University, Chongqing, China; ^2^ Department of Radiology, First Affiliated Hospital of Chongqing Medical University, Chongqing, China; ^3^ Department of Information Center, First Affiliated Hospital of Chongqing Medical University, Chongqing, China; ^4^ Department of Obstetrics and Fetal Medicine Unit, First Affiliated Hospital of Chongqing Medical University, Chongqing, China

**Keywords:** cervical length, massive bleeding, placenta accreta spectrum, placenta previa, cesarean section

## Abstract

**Purpose:**

Abnormal placentation is a spectrum disorder that includes creta, increta, and percreta; the term placenta accreta spectrum (PAS) disorders is used as a broad term to describe all of these conditions. PAS can lead to life-threatening hemorrhage. The predictive value of cervical length (CL) in patients with PAS remains controversial. Thus, this study investigated the relationship between CL and the probability of major bleeding in patients with PAS and placenta previa.

**Methods:**

This retrospective cohort study was conducted at a comprehensive tertiary hospital in Chongqing, China, between January 2018 and December 2020. The target independent and dependent variables were CL and intraoperative massive bleeding, respectively. The covariates included demographic, clinical, and ultrasound characteristics. Logistic regression was used to explore the association between CL and massive bleeding.

**Results:**

In total, 317 participants were enrolled, in whom the prevalence of massive bleeding was 41.9% (133/317). The threshold of CL associated with massive bleeding (≥1,000 ml) was 33 mm based on a receiver operating characteristic curve. In the fully adjusted model for each additional unit of CL, the risk of massive bleeding decreased by 7% [95% confidence interval (CI), 0.88–0.98]. The risk of major bleeding was reduced by 44% in patients with a CL greater than 33 mm (95% CI, 0.33–0.97) compared with patients with a CL less than 33 mm.

**Conclusions:**

CL was negatively associated with massive intraoperative bleeding in patients with PAS combined with placenta previa. When the CL was greater than 33 mm, the risk of bleeding decreased by 44%. Thus, CL can be used as a standalone parameter to identify the risk of massive intraoperative bleeding in patients with suspected PAS.

## Introduction

Abnormal placentation is a spectrum disorder that includes both abnormal adherence (placenta accreta) and abnormal invasion (placenta increta and placenta percreta); thus, the term placenta accreta spectrum (PAS) disorders is used here as a broad term to describe the condition in its entirety (1). The incidence of PAS has gradually increased with an increase in the rate of cesarean deliveries. Elective cesarean delivery remains the predominant treatment modality (2, 3). Intraoperative hemorrhage may lead to adverse obstetric outcomes during cesarean delivery in such patients.

PAS is one of the most commonly understood factors leading to massive hemorrhage (4). However, not all patients with PAS are at risk for major bleeding, and the risk may vary depending on the various forms of PAS. Although imaging is the best investigation method available for prenatal identification of invasive placentation, its sensitivity and specificity are not 100% (5). Some cases of placenta accreta are difficult to diagnose preoperatively, and the preoperative classification of PAS types remains challenging (6).

The ultrasound indicator cervical length (CL) can be easily obtained by transabdominal or vaginal ultrasound. Previous studies have demonstrated that CL can predict preterm labor and antepartum hemorrhage in patients with placenta previa and that a shorter CL is associated with antepartum hemorrhage (7, 8). A shorter CL in placenta previa was also linked to significant intraoperative blood loss, according to previous studies (9–11).

The predictive value of CL in patients with PAS remains controversial. Rac et al. concluded that in patients with placenta accreta, a shorter CL did not increase the likelihood of bleeding or preterm delivery (12). Although CL is associated with PAS, some studies have also indicated that it is not linked to the degree of placental implantation (13). Further research is needed to determine whether there is a link between CL and risk of intraoperative hemorrhage in patients with suspected PAS. In this context, this study aimed to investigate the relationship between CL and the likelihood of major bleeding in patients with PAS and placenta previa.

## Materials and methods

### Study design

This was a retrospective, cohort study. The target independent variable was CL, and the dependent variable was intraoperative massive bleeding. Intraoperative massive bleeding was defined as intraoperative bleeding ≥1,000 ml, and intraoperative bleeding <1,000 ml was considered as non-massive bleeding. Intraoperative blood loss was calculated using preoperative and postoperative hemoglobin values (14). Women with a calculated blood loss of ≥1,000 ml were included in the massive bleeding group, while those with a calculated bleeding volume of <1,000 ml were categorized into the non-massive bleeding group (15).

### Study population

We reviewed the clinical data (extracted from the hospital electronic medical record system) of consecutive patients with PAS with placenta previa who underwent cesarean section (CS) between January 2018 and December 2020. The preoperative diagnosis of PAS was mainly based on the Placenta Accreta Spectrum Ultrasound Scoring System (PASUSS) and (16, 17) FIGO diagnostic criteria. From 2019, the diagnosis of PAS relied primarily on PASUSS. The need for obtaining informed consent was waived because of the retrospective nature of the study. This study was approved by the Ethics Committee of the First Affiliated Hospital of Chongqing Medical University. The inclusion criteria were patients with PAS with placenta previa, identified using color Doppler ultrasonography, and having no other prenatal disorders. The exclusion criteria were patients with additional obstetric diseases, a history of cervical cerclage and other cervical surgeries, and inadequate data.

### Variables

CL was obtained through transabdominal ultrasound or transvaginal ultrasound, performed by an obstetric ultrasound specialized radiologist with 5 years of post-fellowship experience. The shortest value was determined as CL in the sagittal plane. The CL of the uterus was measured from the external os to the functional internal os. CL obtained from the last obstetric ultrasound before cesarean delivery was used as a reference. CL was generally measured on the day before surgery or the day of surgery. The accuracy of CL for identifying women at high risk of massive intraoperative hemorrhage was determined using receiver operating characteristic (ROC) curves. Other variables included (1) continuous variables: age, weight, height, placental thickness, gravidity, and gestational age; (2) categorical variables: curettage, hypervascularization (The degree of post-placental vascularization from visible to very strong is indicated by 1–4, respectively), previous CS, and history of hypertension and diabetes.

### Treatment protocol

The standard procedure for hemorrhage prevention and bleeding control was followed during CS. The surgeon decided whether to perform an aortic balloon placement before cesarean delivery, depending on the patient's condition. When possible, we developed a policy of prophylactic interruption of abdominal aortic balloon catheter placement, followed by placental separation and uterine preservation *via* cesarean delivery. Depending on the operative findings, the decision was made to remove the adherent placenta and apply local sutures in the placental bed or to leave the placenta in place and perform a hysterectomy after the newborn was delivered. The Triple-*P* procedure was used in some patients to preserve the uterus as much as possible.

### Follow-up procedure

We performed the follow-up until the patient was discharged from the hospital. Follow-up data were stored in the hospital's electronic medical record system.

### Statistical analysis

Continuous data are presented as the mean ± standard deviation or median values, depending on the normality of distribution. Normality was examined using the Shapiro–Wilk test. The t-test was used for continuous variables with a normal distribution. Categorical data are presented as the number of cases and corresponding percentage. Categorical and continuous data that did not show a normal distribution were analyzed using Pearson's chi-squared test, Fisher's exact test, or Mann–Whitney U test, as appropriate.

Univariate and multivariate binary logistic regression analyses were performed with four constructed models: Model 1, no covariates adjusted; Model 2, only adjusted for sociodemographic data; Model 3, adjusted for covariates presented in Table 1; and Model 4, adjusted for all variables in Model 2 + Model 3.

**Table 1:** Baseline characteristics of the study participants.

Variables	Total (*n* = 317)	CL < 33 (*n* = 140)	CL ≥ 33 (*n* = 177)	*p*
Age (year), Mean ± SD	32.9 ± 4.3	32.8 ± 4.4	33.0 ± 4.2	0.649
Weight (kg), Mean ± SD	68.4 ± 8.8	68.2 ± 8.9	68.6 ± 8.8	0.678
Height (cm), Mean ± SD	157.9 ± 4.8	157.9 ± 4.4	157.8 ± 5.2	0.860
Gestational age (week)	36.3 ± 1.6	36.2 ± 1.7	36.5 ± 1.5	0.116
Gravidity, Mean ± SD	4.0 (3.0, 5.0)	4.0 (3.0, 5.0)	4.0 (2.0, 5.0)	0.621
Curettage, *n* (%)	2.0 (1.0, 3.0)	1.0 (1.0, 2.0)	2.0 (1.0, 3.0)	0.179
Previous CS, *n* (%)				0.173
≤1	268 (84.5)	114 (81.4)	154 (87)	
≥2	49 (15.5)	26 (18.6)	23 (13)	
Hypervascularization, *n* (%)				<0.001
1	108 (34.1)	31 (22.1)	77 (43.5)	
2	119 (37.5)	59 (42.1)	60 (33.9)	
3	67 (21.1)	37 (26.4)	30 (16.9)	
4	23 (7.3)	13 (9.3)	10 (5.6)	
Placental thickness (mm), Mean ± SD	42.0 ± 9.0	43.6 ± 9.1	40.8 ± 8.8	0.006
Suspected PAS, *n* (%)				<0.001
No PAS	18 (5.7)	2 (1.4)	16 (9)	
Creta	103 (32.5)	29 (20.7)	74 (41.8)	
Increta	170 (53.6)	91 (65)	79 (44.6)	
Percreta	26 (8.2)	18 (12.9)	8 (4.5)	
Diabetes, *n* (%)	83 (26.2)	44 (31.4)	39 (22)	0.059
Hypertension, *n* (%)	11 (3.5)	3 (2.1)	8 (4.5)	0.358

CL, cervical canal length; CS, cesarean section; SD, standard deviation; PAS, placenta accreta spectrum.

Stratified binary logistic regression models were used for subgroup analyses. We performed an interaction test after converting the continuous variables into categorical variables based on the clinical cut-off point. The likelihood ratio test was followed by tests for the effect modification of subgroup indicators. The purpose of converting CL into a categorical variable was to validate the results of CL as a continuous variable.

All the analyses were performed with the statistical software packages R 3.3.2 (http://www.R-project.org, The R Foundation) and Free Statistics software version 1.6. A two-tailed test was performed. *P* values less than 0.05 (two-sided) were considered statistically significant.

## Results

**Figure 1:**
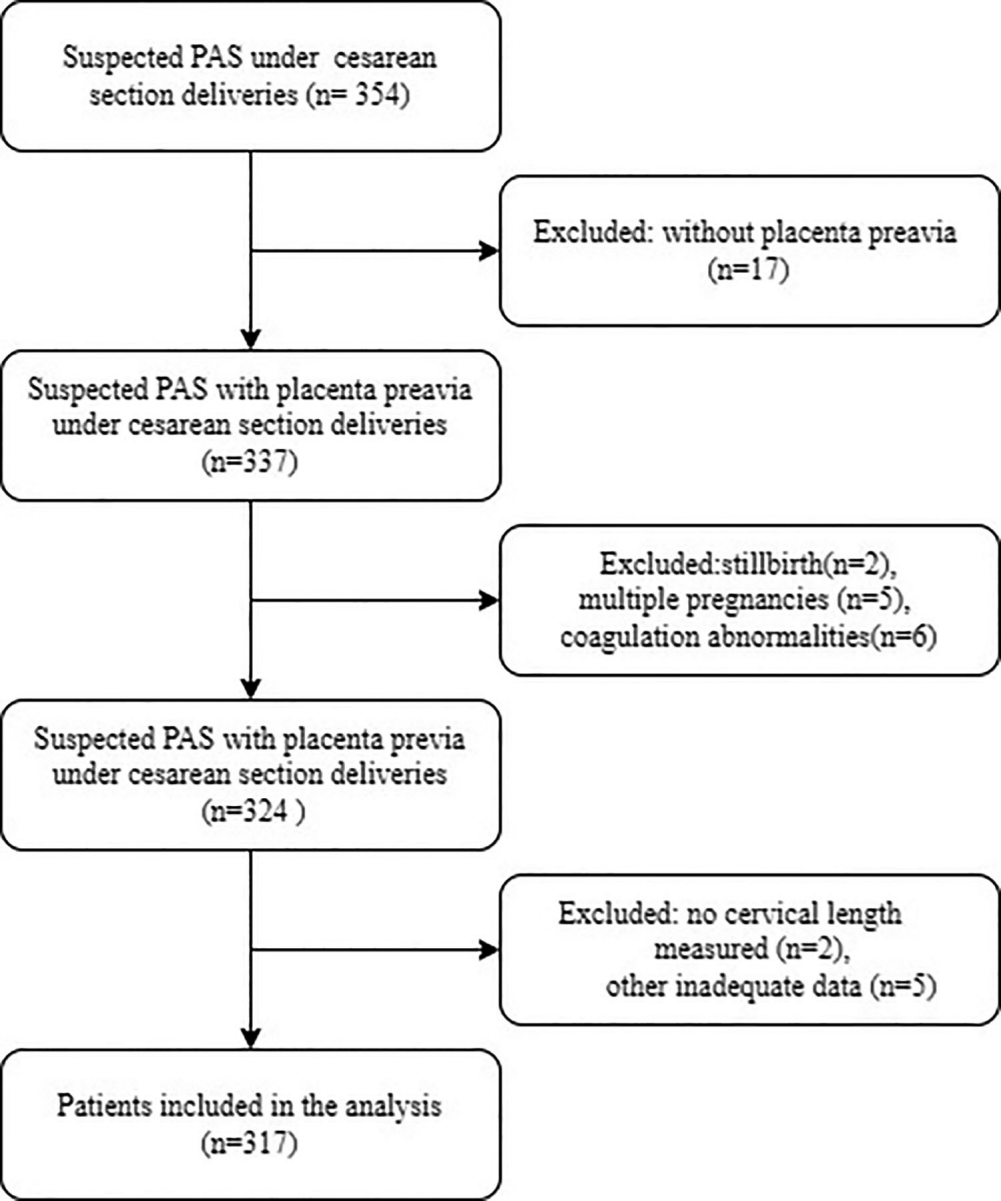
Flowchart showing the study population selection.

A total of 317 participants were enrolled for the final data analysis (Figure 1 presents a flowchart of patient selection). The baseline characteristics of the selected participants are shown in Table 1, according to the optimal cut-off value based on the ROC of CL. Participants in the lower CL group had a greater volume of blood loss, longer operation and hospitalization time, and higher incidence of intra-abdominal balloon occlusion (IABO) and blood transfusion than those in the higher CL group (Table 2).

**Table 2:** Intraoperative and postoperative data of the study participants.

Variables	Total (*n* = 317)	CL < 33 (*n* = 140)	CL ≥ 33 (*n* = 177)	*p*
Blood loss (mL), Median (IQR)	611.0 (436.0, 1124.0)	1002.0 (464.2, 1440.0)	566.0 (422.0, 1023.0)	0.003
IABO, *n* (%)	168 (53.0)	99 (70.7)	69 (39)	<0.001
Blood transfusion, *n* (%)	78 (24.6)	42 (30)	36 (20.3)	0.047
Postoperative transfusion, *n* (%)	60 (18.9)	36 (25.7)	24 (13.6)	0.006
Plasma, *n* (%)	55 (17.4)	31 (22.1)	24 (13.6)	0.045
Cryoprecipitate, *n* (%)	18 (5.6)	10 (7.1)	8 (4.6)	0.653
Autologous Blood Collection, *n* (%)	43 (13.6)	25 (17.9)	18 (10.2)	0.047
Uterine gauze stuffing, *n* (%)	45 (14.2)	25 (17.9)	20 (11.3)	0.097
Intrauterine balloon tamponade, *n* (%)	111 (35.0)	53 (37.9)	58 (32.8)	0.346
Uterine artery embolization, *n* (%)	25 (7.9)	14 (10)	11 (6.2)	0.214
Uterine Bondage, *n* (%)	141 (44.5)	67 (47.9)	74 (41.8)	0.282
Cervical lift suture, *n* (%)	226 (71.3)	108 (77.1)	118 (66.7)	0.041
Triple-*P* procedure, *n* (%)	58 (18.3)	29 (20.7)	29 (16.4)	0.322
Placenta left *in situ*, *n* (%)	21 (6.6)	12 (8.6)	9 (5.1)	0.215
Hysterectomy, *n* (%)	11 (3.5)	7 (5)	4 (2.3)	0.224
ICU admission, *n* (%)	12 (3.8)	5 (3.6)	7 (4)	0.859
Operation time (min), Median (IQR)	103.0 (85.0, 128.0)	111.5 (92.5, 142.2)	95.0 (80.0, 120.0)	< 0.001
Hospitalization time (day), Median (IQR)	6.0 (5.0, 7.0)	6.0 (5.0, 8.0)	5.0 (5.0, 7.0)	0.009
Neonatal weight (g), Mean ± SD	2880.9 ± 471.3	2823.3 ± 451.8	2926.5 ± 482.6	0.053
Neonatal Sex, *n* (%)				0.711
Male	169 (53.3)	73 (52.1)	96 (54.2)	
Female	148 (46.7)	67 (47.9)	81 (45.8)	

CL, cervical canal length; IABO, intra-abdominal balloon occlusion; ICU, intensive care unit; IQR, interquartile range; SD, standard deviation.

The results of univariate analyses are shown in Supplementary Table S1. Univariate binary logistic regression analysis revealed that age, height, and weight were not associated with massive intraoperative bleeding. We further discovered that CL was negatively associated with massive intraoperative bleeding. In contrast, univariate analysis showed that placental thickness, previous CS, hypervascularization, and curettage were positively correlated with massive intraoperative bleeding.

In this study, we constructed four models to analyze the independent effects of CL on massive intraoperative bleeding (univariate and multivariate binary logistic regression analyses). The effect sizes [odds ratios (OR)] and 95% confidence intervals (CI) are listed in Table 3. In the unadjusted model (Model 1), the model-based effect size could be explained as the difference in one unit of CL associated with a 10% reduction in the risk of massive bleeding. In the minimum-adjusted model (Model 2), as the CL increased by one unit, the risk of massive bleeding decreased by 10% (95% CI, 0.86–0.95). In the fully adjusted model (Model 4), for each additional unit of CL, the risk of massive bleeding decreased by 7% (95% CI, 0.88–0.98). For sensitivity analysis, we converted the CL from a continuous variable to a categorical variable (optimal cut-off value based on ROC), and after adjusting for all variables, the risk of major bleeding was reduced by 44% in patients with a CL greater than 33 mm (95% CI, 0.33–0.97), compared with that in patients with a CL less than 33 mm.

**Table 3:** Multivariate analysis for massive bleeding.

Outcome	Non-adjusted Model		Model I		Model II		Model III	
	OR (95% CI)	*P*	OR (95% CI)	*P*	OR (95% CI)	*P*	OR (95% CI)	*P*
Cervical length	0.9 (0.86–0.95)	<0.001	0.9 (0.86-0.95)	<0.001	0.93 (0.88∼0.99)	0.016	0.93 (0.88∼0.98)	0.011
Cervical length classification								
<33 mm	Reference		Reference		Reference		Reference
≥33 mm	0.45 (0.28∼0.7)	0.001	0.44 (0.28∼0.7)	<0.001	0.55 (0.32∼0.95)	0.032	0.56 (0.33∼0.97)	0.039

Model I: Adjust for age, weight, and height.

Model II: Adjust for hypervascularization prior to CS, gravidity, gestational age, placental thickness, curettage, and IABO.

Model III: Adjusted for age, weight, height, hypervascularization, previous CS, gravidity, gestational age, placental thickness, curettage, and IABO.

OR, odds ratio; CI, confidence interval; CS, cesarean section; IABO, intra-abdominal balloon occlusion.

**Figure 2:**
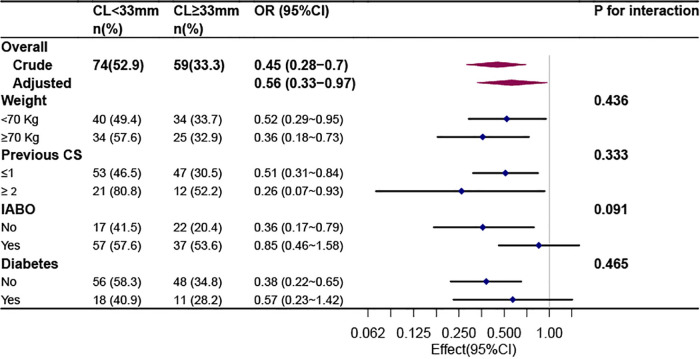
Stratified analyses of the association between cervical length and massive bleeding according to baseline characteristics.

We selected body weight, previous CS, history of diabetes, and incidence of IABO as stratification variables to study the trend of effect sizes for these variables (Figure 2); however, no significant interaction was observed (*P* > 0.05).

## Discussion

Our findings indicated that CL was negatively associated with massive intraoperative bleeding after adjusting for other covariates. A shorter CL was associated with a higher risk of massive bleeding, independent of other ultrasound and clinical indicators. In addition, when the CL was greater than 33 mm, the risk of bleeding decreased by 44% (95% CI, 0.33–0.97). Thus, CL could be used as a standalone parameter to aid in identifying the risk of massive intraoperative bleeding in patients with suspected PAS.

Similar findings have been reported in previous studies. In a previous study on patients with placenta previa, bleeding is negatively correlated with CL (8). As shown on the ROC curve, the threshold CL associated with major bleeding (>2500 ml) was 25 mm. The relative risk for massive bleeding in cases with a short CL (<25 mm) was 7.2 (95% CI, 2.3–22.3) in comparison to that in cases with a long CL (8). A short CL was also associated with poor maternal outcomes (18, 19). Fukushima et al. reported that in placenta previa, a CL ≤ 30 mm was associated with placental adhesions and massive intraoperative blood loss (9). A short cervix, especially shorter than 20.5 mm, may make the surgery more difficult and lead to increased bleeding (10). CL predicts surgical outcomes, and a shorter CL is associated with the need to perform a hysterectomy (11).

Most of the previous studies have focused on patients with placenta previa or hypoplacenta. Our findings support a similar relationship in patients with PAS. Although PAS is a risk factor for postpartum hemorrhage, our study found intraoperative hemorrhage in only a subset of patients, particularly after preoperative intervention or various intraoperative hemostatic measures. Furthermore, we confirmed a specific relationship between CL and intraoperative bleeding in patients with PAS. Using CL as a parameter, we were able to determine which patients with suspected PAS were more likely to have intraoperative hemorrhage.

Several hypotheses have been proposed to explain why people with short cervixes tend to bleed intraoperatively. First, most postpartum bleeding is caused by weak uterine contractions (20), which may be exacerbated in patients with a shortened cervix. Furthermore, a shorter cervix suggests that more cervical components are involved in the composition of the lower uterine segment, and fewer uterine muscles may affect lower uterine segment contraction, which is one of the reasons why these contractions are less intense (8, 21). When placental abruption occurs in the lower uterine segment, the muscle layer of this segment is unable to contract the torn blood vessels (22). Second, in patients with PAS combined with placenta previa, contraction of the lower uterine segment may be limited in the context of placental implantation (8). Third, shortening of the cervix makes surgical operations involving hemostasis more difficult and can prolong the time to hemostasis. A potentially effective method of controlling severe postpartum hemorrhage due to placenta previa/implanted placenta previa is turning the cervix into the uterine cavity and suturing the anterior and/or posterior cervical lips to the anterior and/or posterior wall of the lower uterine segment, respectively (23). In patients with focal placental implantation having a low fetal count and desiring future fertility, the use of the cervix as a tamponade combined with bilateral uterine artery ligation appears to be a safe alternative to hysterectomy (24). Fourth, patients with a shortened cervix have an increased probability of antepartum bleeding, which may deplete some clotting factors. As the time to hemostasis increases, excessive depletion of clotting factors can further increase the difficulty to achieve hemostasis, although this hypothesis needs to be confirmed by further studies. Fifth, a short cervix may present differences in placental blood supply compared to a long cervix, leading to increased difficulty in stopping bleeding and increased surgical difficulty (13).

This study has some limitations. First, some limitations inherent to its retrospective design, such as confounders and selection bias. Second, this was a retrospective study; therefore, CL was not measured prospectively. Instead, the CL measurements were obtained by transabdominal and transvaginal ultrasound, which may have affected the final result. The measurement of CL by different physicians may lead to biased results, although all of these physicians have extensive clinical experience and the CL obtained had good reproducibility, which should have limited the differences (25). Third, the CS procedure was performed by different surgeons with different choices or preferences, which may affect the final amount of intraoperative bleeding, even though all surgeons have the ultimate goal of stopping bleeding and protecting the uterus as much as possible. Fourth, all ultrasound-related metrics were obtained by reviewing the patients' ultrasound reports; therefore, some ultrasound metrics could not be included in the reports. Consequently, some relevant indicators were excluded from the regression model. All of these indicators were potential confounding factors that could affect the final results.

In conclusion, CL is negatively associated with massive intraoperative bleeding in patients with PAS combined with placenta previa. This objective parameter will be easy to use even for non-expert imaging technicians for screening and will lead to an appropriate referral to centers of excellence for PAS disorders. When the CL was greater than 33 mm, the risk of bleeding decreased by 44% (95% CI, 0.33–0.97). Thus, CL can be used as a standalone parameter to aid in identifying the risk of massive intraoperative bleeding in women with suspected PAS.

## Data Availability

The raw data supporting the conclusions of this article will be made available by the authors, without undue reservation.
