# Supplementary material for: Association between cervical length and massive intraoperative bleeding in patients with suspected placenta accreta spectrum combined with placenta previa: A retrospective cohort study
Source: Front Surg. 2022 Oct 21;9:1028494. doi: 10.3389/fsurg.2022.1028494 (PMC9634406; doi:10.3389/fsurg.2022.1028494)
Supplement: Supplementary file 1 [file Table1.docx]

Supplementary Table 1. Univariate analysis for massive bleeding.

| Variable | OR_95CI | *P* |
| --- | --- | --- |
| Cervical canal length | 0.89 (0.85~0.94) | <0.001 |
| Placental thickness | 1.06 (1.03~1.09) | <0.001 |
| Previous CS ≥2 | 3.46 (1.82~6.61) | <0.001 |
| hypervascularization2 | 4.6 (2.45~8.65) | <0.001 |
| hypervascularization3 | 8.99 (4.39~18.41) | <0.001 |
| hypervascularization4 | 25.43 (7.69~84.09) | <0.001 |
| Gestational age | 0.82 (0.7~0.95) | 0.01 |
| curettage1 | 1.45 (0.74~2.83) | 0.273 |
| curettage2 | 2.3 (1.18~4.49) | 0.015 |
| curettage ≥3 | 1.56 (0.8~3.04) | 0.193 |
| gravidity | 1.24 (1.09~1.42) | 0.001 |
| Age | 1 (0.95~1.05) | 0.962 |
| Weight | 1.01 (0.98~1.04) | 0.478 |
| Height | 0.99 (0.95~1.04) | 0.7 |
| IABO | 3.58 (2.23~5.76) | <0.001 |

CI, confidence interval; CS, Cesarean section IABO, Intra-Abdominal Balloon Occlusion.
